# Vitamin B_12_ Is Associated with Higher Serum Testosterone Concentrations and Improved Androgenic Profiles Among Men with Infertility

**DOI:** 10.1016/j.tjnut.2024.06.013

**Published:** 2024-06-26

**Authors:** Matineh Rastegar Panah, Keith Jarvi, Kirk Lo, Ahmed El-Sohemy

**Affiliations:** 1 Department of Nutritional Sciences, University of Toronto, Toronto, Canada; 2 Department of Urology, Murray Koffler Urologic Wellness Center, Mount Sinai Hospital, Toronto, Canada

**Keywords:** male infertility, micronutrients, vitamin B_12_, reproductive hormones, testosterone

## Abstract

**Background:**

Infertility impacts 16% of North American couples, with male factor infertility contributing to ∼30% of cases. Reproductive hormones, especially testosterone, are essential for spermatogenesis. An age-independent population-level decline in testosterone concentrations over the past few decades has been proposed to be a consequence of diet and lifestyle changes. Vitamin B_12_ is present in the testes and has been suggested as an adjuvant nutritional therapy for male infertility due to its potential to improve sperm parameters. However, evidence examining the relationship between vitamin B_12_ and reproductive hormones is limited.

**Objectives:**

The objective was to cross-sectionally examine the relationship between serum vitamin B_12_ and male reproductive hormones (luteinizing hormone, follicular stimulating hormone, total testosterone, estradiol, and prolactin).

**Methods:**

Men with infertility (*n* = 303) were recruited from Mount Sinai Hospital in Toronto, Canada. Serum was analyzed for vitamin B_12_ and reproductive hormones. Statistical analyses included nonparametric Spearman’s rank correlation coefficient, linear regression, logistic regression, and effect modification by age and BMI linear regressions.

**Results:**

An independent monotonic relationship between serum vitamin B_12_ and total testosterone (ρ = 0.19, *P* = 0.001) was observed. Serum vitamin B_12_ was linearly associated with total testosterone (unadjusted β = 0.0007, *P* = 0.008 and adjusted β = 0.0005, *P* = 0.03). Compared to individuals in the lowest tertile of serum vitamin B_12_, those in the middle tertile (adjusted odds ratio [OR] = 0.48; 95% confidence interval [CI]: 0.25, 0.93, *P* = 0.03) and the highest tertile (unadjusted OR = 0.41; 95% CI: 0.22, 0.77, *P* = 0.005 and adjusted OR = 0.44; 95% CI: 0.22, 0.87, *P* = 0.02) had reduced odds of testosterone deficiency.

**Conclusions:**

These findings suggest that among men with infertility, low serum vitamin B_12_ is associated with a higher risk of testosterone deficiency and impaired androgenic hormonal profiles that impact spermatogenesis and consequently, fertility.

## Introduction

Infertility is characterized as the inability to achieve clinical pregnancy after a year of consistent unprotected sexual intercourse between a man and woman assigned to those genders at birth. Approximately 16% of couples in North America are affected by infertility, with male factor infertility accounting for ∼30% of cases and a combination of male and female factors contributing to another 20% of cases [1]. The decline in male fertility, characterized by factors such as diminished sperm quality and hypogonadism, has become a growing concern [2,3]. Previous research has identified a significant decrease in testosterone concentrations among males, independent of age, attributed to various influences including health, environmental factors, and dietary choices [2,4]. Although endocrine dysfunctions, physical impairments, and genetic polymorphisms were traditionally considered primary causes of male infertility, recent studies have shed light on the role of lifestyle factors such as sleep disruptions, body weight, smoking, exposure to environmental toxins, and nutrition [[5], [6], [7], [8], [9], [10], [11], [12], [13], [14], [15]]. Promising evidence suggests that adopting a dietary pattern, characterized by a higher consumption of fruits and vegetables while limiting meat, fat, refined sugars, and processed foods, can positively impact sperm parameters [[16], [17], [18], [19]]. Vitamin B_12_ has gained attention for its potential impact on male reproductive function, as highlighted in a recent review investigating the influence of nutrition and genetics on male fertility [20].

Seminal parameters and reproductive hormones are commonly used as indicators of male fecundity and for diagnosing male factor infertility [21,22]. Although existing research in the field of nutrition and male fertility primarily focuses on seminal parameters, there is a significant knowledge gap regarding the influence of micronutrients on male reproductive hormones, which play a crucial role in spermatogenesis and overall male reproductive health. The hypothalamic–pituitary–gonadal (HPG) axis serves as the principal signaling pathway responsible for regulating reproductive hormones, thus overseeing spermatogenesis [23]. Within this HPG axis, the hypothalamus releases gonadotropin-releasing hormone, which stimulates the secretion of gonadotropins, follicular stimulating hormone (FSH), and luteinizing hormone (LH) [23]. FSH primarily acts on Sertoli cells in the testes, supporting spermatogenesis and the maturation of sperm cells, whereas LH stimulates Leydig cells to produce intratesticular androgens, including testosterone, which is essential for spermatogenesis [23]. Androgens can also be converted into estrogens, particularly estradiol, in the testes and peripheral tissues through the action of aromatase (CYP19) [24]. Elevated concentrations of estradiol can potentially hinder fertility [25]. Another hormone pertinent to male reproductive health is prolactin, which inhibits the HPG axis, leading to reduced testosterone synthesis [26].

Vitamin B_12_ (cobalamin), is an essential water-soluble vitamin critical to several physiological functions such as DNA synthesis and red blood cell maturation. Some studies have suggested vitamin B_12_ as adjuvant nutritional therapy for male infertility due to its potential to improve sperm health [20,27]. Methylcobalamin, the active form of vitamin B_12_, plays a critical role in homocysteine’s remethylation cycle [28]. It acts as an essential cofactor for methionine synthase, facilitating the transfer of a methyl group from 5-methyltetrahydrofolate to homocysteine in the conversion pathway from homocysteine to methionine [28]. Hyperhomocystenenmia, often attributable to low vitamin B_12_ and folate concentrations, has been associated with oxidative stress and diminished fertility parameters including increased DNA fragmentation and reduced successful in vitro fertilization, reduced sperm concentration, and diminished sperm motility [18,[29], [30], [31]]. A review by Banihani et al. [27] on vitamin B_12_ summarized some limited existing research, which demonstrated a positive association between vitamin B_12_ and improved sperm count, sperm motility, and DNA integrity. Such findings can be attributable to the influence of vitamin B_12_ on homocysteine, enhanced control of nuclear factor-κB, decreased spermatozoa energy production, improved reproductive organ functionality, decreased nitric oxide production, and reduced inflammation-induced sperm impairments [27]. However, there is contrary evidence from other observational studies and clinical trials that have found no association between vitamin B_12_ and semen quality [[32], [33], [34]]. For example, a cross-sectional study by Jathar et al. [34] found that although mean seminal plasma vitamin B_12_ concentrations were lower in individuals belonging to the azoospermic group compared with individuals belonging to the normospermic and oligospermic group, seminal plasma vitamin B_12_ concentrations were not associated with sperm parameters, including, sperm count, motility, or morphology. Only one study involving a small group of 26 males with infertility has examined the relationship between vitamin B_12_ and reproductive hormones [35]. A comprehensive review on vitamin B_12_ and male reproductive health, highlighted this scarcity in research on the impact of vitamin B_12_ on reproductive hormones [27]. Therefore, the objective of the present study was to assess the association between serum vitamin B_12_ concentrations and reproductive hormones in a population of men experiencing infertility.

## Methods

### Study design and participants

This study employed a cross-sectional design to assess the relationship between serum vitamin B_12_ concentrations and reproductive hormones. A cohort of 832 males experiencing infertility was recruited with informed consent, from the Murray Koffler Urologic Wellness Center at Mount Sinai Hospital in Toronto, Canada. The recruitment phase from June 2019 to August 2021, utilized a clinic-based recruitment strategy where participants were enrolled as they attended their clinic appointments. Eligibility criteria excluded individuals who had undergone postoperative procedures; experienced physical impairments leading to infertility; were unable to provide venous blood or semen samples; had missing data; had recently used fertility-related medication within the last 6 mo, had undergone vasectomy; received testicular cancer radiation therapy <4 y ago; or had conditions such as Klinefelter Syndrome, Y chromosome microdeletions, or cystic fibrosis. After applying these exclusions, the final sample size consisted of 303 males (Supplemental Figure 1). Bloodwork and hormonal assays were missing at random due to challenges with accessing laboratory services during the COVID-19 pandemic. The present study adhered to ethical standards set by the University of Toronto Research Ethics Board and Mount Sinai Hospital Research Ethics Board. Approval was obtained from these relevant committees overseeing research involving human participants. All participants provided written informed consent to participate in the study.

### Anthropometric measurements and general health information

Participants completed a computerized personal health questionnaire provided by Mount Sinai Hospital’s Men’s Health Institute. The questionnaire gathered information on anthropometric measurements, demographic details, medical history, lifestyle data, and female partner medical history. Further information regarding the specific components and details of the questionnaire have been previously described [36].

### Nutritional and reproductive hormone biochemical measurements

Serum vitamin B_12_ and reproductive hormone concentrations were assessed by collecting venous blood samples from the participants. The analyses were conducted at either the onsite laboratory of Mount Sinai Hospital or at LifeLabs medical laboratory services. The reproductive hormones included in the analyses were FSH, LH, total testosterone (TT), prolactin, and estradiol. These measurements were conducted using ELISA assays in accordance with the laboratory’s standard operating procedures. Estradiol concentrations below 93 pmol/L were reported as values < 93 pmol/L by the laboratory.

### Statistical analyses

The statistical software R Studio (Version 1.1.463) was utilized for the data analyses. Statistical significance was defined as *P* < 0.05. Descriptive statistics, including counts and the corresponding percentages relative to the total sample as well as mean (±SD), were used to summarize subject characteristics with categorical and continuous variables. Differences between the tertiles of serum vitamin B_12_ groups were assessed using chi-square test and analysis of variance for categorical variables and continuous variables, respectively.

The monotonic relationship between serum vitamin B_12_ concentrations and reproductive hormones, excluding estradiol, was evaluated using nonparametric Spearman’s rank correlation coefficient analyses. The Shapiro–Wilk test was employed to examine normality. For parametric analyses, square root transformation was applied to reproductive hormones to normalize skewed distributions. Residual distributions of all models were assessed for normality and variance inflation factor was computed to assess for collinearity. For initial parametric analyses, linear regression models were used to assess the association between serum vitamin B_12_ concentrations and reproductive hormones as continuous variables. Both unadjusted and covariate-adjusted linear regression analyses were completed. To explore potential effect modification, additional linear regression models were constructed by incorporating interaction terms for age and serum vitamin B_12_ concentrations, as well as for BMI and serum vitamin B_12_ concentrations. For any model(s) showing significant interactions, simple slope and false discovery rate-corrected Johnson–Neyman interval analyses were conducted.

Further analyses were completed where logistic regression models were employed, categorizing serum vitamin B_12_ concentration into tertiles and dichotomizing reproductive hormones into clinically significant categories. Serum vitamin B_12_ concentration was grouped into tertiles instead of being categorized based on deficiency and elevated concentration cutoffs. This approach was selected because deficiency and elevated clinical cutoffs for serum vitamin B_12_ are established based on disease-related epidemiological data and markers unrelated to fertility. The clinical cutoffs for reproductive hormones were determined based on values outside their respective normal ranges: elevated FSH (>12.4 IU/L), elevated LH (>7.8 IU/L), low TT (<9.2 nmol/L), elevated prolactin (>18 ng/mL), and elevated estradiol (>146.8 pmol/L). In the multivariate linear and logistic regression models, the clinically relevant covariates adjusted for were age (y), BMI (kg/m^2^), current alcohol consumption (yes/no/unspecified), current smoking status (yes/no), meteorological season blood was drawn (categorized as winter, spring, summer, and fall), and ethnicity (Caucasian, African-Canadian, Asian, Hispanic, Indo-Canadian, Middle-Eastern, and unspecified).

## Results

Table 1 presents the descriptive characteristics of the study participants. The mean ± SD concentration of serum vitamin B_12_ was 446 (±203) pmol/L, with 0.7% of the population having vitamin B_12_ deficiency (<148 pmol/L) and 12.2% exhibiting elevated vitamin B_12_ concentrations (>701 pmol/L). For reproductive hormones, the mean FSH, LH, TT, and prolactin concentration was 9.7 (±9.4) IU/L, 7.2 (±5.0) IU/L, 13.3 (±6.7) nd/dL, and 8.8 (±3.7) ng/mL, respectively. Furthermore, in terms of reproductive hormones outside the normal range, 24.8% of participants had elevated FSH, 33.7% had elevated LH, 30% had low testosterone, 2.6% showed elevated prolactin concentrations, and 74.9% displayed elevated estradiol concentrations. The mean age of the participants was 36.5 (±5.8) y, and the average BMI was 28.0 (±5.8) kg/m^2^. Among the participants, 27.7% (*n* = 84) fell within the normal weight category (BMI < 25 kg/m^2^), 48.2% (*n* = 146) were classified as overweight (BMI, 25–29.9 kg/m^2^), and 24.1% (*n* = 73) were within the obese category (BMI ≥ 30 kg/m^2^), with corresponding mean BMIs of 22.9 ± 1.5, 27.2 ± 1.3, and 35.6 ± 6.6 kg/m^2^, respectively. The majority of participants identified as Caucasian (43.9%), followed by Asian (19.1%), unspecified (15.2%), African-Canadian (8.9%), Middle-Eastern (6.3%), Indo-Canadian (4.3%), and Hispanic (2.3%). For lifestyle factors, the majority of participants were nonsmokers (86.5%) and reported not consuming alcohol (53.1%). Analyzing the data using analysis of variance and chi-square tests, we observed significant variations across serum vitamin B_12_ tertile groups for age (*P* = 0.03), ethnicity (*P* = 0.03), serum TT (*P* = 0.009), and TT clinical status (*P* = 0.01).

**TABLE 1:** Subject characteristics.

Characteristics	*N* (%)	Mean ± SD	Tertiles of serum vitamin B_12_^1^			*P*^1^
			Lowest tertile	Mid-tertile	Highest tertile	
Serum vitamin B_12_ biomarkers						
Count, *n* (%)			102 (33.7)	100 (33.0)	101 (33.3)	
Serum vitamin B_12_ (pmol/L)		446 ± 203	268 ± 51.3	401 ± 37.8	670 ± 189.0	
Vitamin B_12_ clinical status^3^, *n* (%)						
Deficient	2 (0.7)					
Optimal	264 (87.1)					
Elevated	37 (12.2)					
Factors of clinical relevance						
Age (y), mean ± SD		36.5 ± 5.8	36.1 ± 6.1	35.7 ± 4.96	37.7 ± 6.01	0.03
BMI (kg/m^2^), mean ± SD		28.0 ± 5.8	28.3 ± 6.1	28.3 ± 6.8	27.5 ± 4.2	0.55
Current smoker, *n* (%)						0.13
No	262 (86.5)		86 (84.3)	83 (83.0)	93 (92.1)	
Yes	41 (13.5)		16 (15.7)	17 (17.0)	8 (7.9)	
Alcohol consumption, *n* (%)						0.66
No	161 (53.1)		58 (56.9)	53 (53.0)	50 (49.5)	
Yes	136 (44.9)		43 (42.2)	45 (45.0)	48 (47.5)	
Unspecified	6 (2.0)		1 (1.0)	2 (2.0)	3 (3.0)	
Ethnicity, *n* (%)						0.03
Caucasian	133 (43.9)		44 (43.1)	37 (37.0)	52 (51.5)	
Asian	58 (19.1)		24 (23.5)	23 (23.0)	11 (10.9)	
Unspecified	46 (15.2)		16 (15.7)	16 (16.0)	14 (13.9)	
African-Canadian	27 (8.9)		7 (6.9)	5 (5.0)	15 (14.9)	
Middle-Eastern	19 (6.3)		5 (4.9)	11 (11.0)	3 (3.0)	
Indo-Canadian	13 (4.3)		5 (4.9)	4 (4.0)	4 (4.0)	
Hispanic	7 (2.3)		1 (1.0)	4 (4.0)	2 (2.0)	
Seasonal variation^4^, *n* (%)						0.18
Fall	97 (32.0)		34 (33.3)	25 (25.0)	38 (37.6)	
Winter	90 (29.7)		33 (32.4)	36 (36.0)	21 (20.8)	
Summer	85 (28.1)		27 (26.5)	30 (30.0)	28 (27.7)	
Spring	31 (10.2)		8 (7.8)	9 (9.0)	14 (13.9)	
Reproductive hormones						
Serum FSH (IU/L)		9.7 ± 9.4	11.2 ± 11.1	8.79 ± 8.63	9.13 ± 8.12	0.13
Serum LH (IU/L)		7.2 ± 5.0	8.07 ± 5.27	6.96 ± 5.25	6.66 ± 4.29	0.10
Serum TT (ng/dL)		13.3 ± 6.7	11.7 ± 6.00	13.6 ± 6.90	14.6 ± 6.78	0.009
Serum prolactin (ng/mL)		8.8 ± 3.7	8.50 ± 3.37	9.37 ± 4.09	8.40 ± 3.48	0.12
FSH clinical status^5^, *n* (%)						0.26
Normal	228 (75.2)		71 (69.6)	79 (79.0)	78 (77.2)	
Elevated	75 (24.8)		31 (30.4)	21 (21.0)	23 (22.8)	
LH clinical status^5^, *n* (%)						0.06
Normal	201 (66.3)		59 (57.8)	68 (68.0)	74 (73.3)	
Elevated	102 (33.7)		43 (42.2)	32 (32.0)	27 (26.7)	
TT clinical status^5^, *n* (%)						0.01
Normal	212 (70.0)		61 (59.8)	72 (72.0)	79 (78.2)	
Low	91 (30.0)		41 (40.2)	28 (28.0)	22 (21.8)	
Prolactin clinical status^5^, *n* (%)						0.40
Normal	295 (97.4)		101 (99.0)	96 (96.0)	98 (97.0)	
Elevated	8 (2.6)		1 (1.0)	4 (4.0)	3 (3.0)	
Estradiol clinical status^5^, *n* (%)						0.62
Elevated	227 (74.9)		3 (71.6)	76 (76.0)	78 (77.2)	
Normal	76 (25.1)		29 (28.4)	24 (24.0)	23 (22.8)	

Abbreviations: BMI, body mass index; FSH, follicular stimulating hormone; LH, luteinizing hormone; SD, standard deviation; TT, total testosterone.

1 Tertile of serum vitamin B_12_ concentration cutoffs are ≤340 pmol/L, >340 to <472.7 pmol/L, and ≥472.7 pmol/L for low, mid-, and highest tertile, respectively.

2 Differences between groups were compared using chi-square for categorical variables and analysis of variance for continuous variables.

3 Serum vitamin B_12_ concentration cutoffs are <148 pmol/L, ≥148 to <701 pmol/L, and ≥701 pmol/L for deficient, optimal, and elevated, respectively.

4 Seasonal variation was determined based on meteorological seasons blood was drawn for analysis, “Winter” = December 1 to February 28/29; “Spring” = March 1 to May 31; “Summer” = June 1 to August 31; “Fall” = September 1 to November 30.

5 Clinical status cutoffs were based on reproductive hormones not within normal range: elevated FSH (>12.4 IU/L), elevated LH (>7.8 IU/L), low TT (<9.2 nmol/L), elevated prolactin (>18 ng/mL), and elevated estradiol (>146.8 pmol/L).

Table 2 presents the results of the nonparametric Spearman’s rank correlation coefficient analyses, revealing a statistically significant and independent monotonic positive association between serum vitamin B_12_ concentrations and TT (ρ = 0.19, *P* = 0.001). Spearman’s rank correlation coefficient analyses indicate that higher vitamin B_12_ concentrations were associated with higher TT concentrations. No associations were observed between vitamin B_12_ concentrations and all remaining reproductive hormones (LH, FSH, and prolactin).

**TABLE 2:** Spearman correlations for the independent association between serum vitamin B_12_ concentration and reproductive hormone concentrations.

Reproductive hormones	ρ	*P*
FSH	−0.07	0.24
LH	−0.09	0.12
TT	0.19	0.001
Prolactin	0.003	0.95
Estradiol^1^	—	—

Abbreviations: FSH, follicular stimulating hormone; LH, luteinizing hormone; TT, total testosterone.

1 Due to the limited sensitivity of the laboratory measurement, estradiol was categorized into 2 groups based on its concentration: normal (≤146.8 pmol/L) and elevated (>146.8 pmol/L). As a result, Spearman’s rank correlation test could not be performed for estradiol.

Assessment of the linear relationship between serum vitamin B_12_ concentrations and square root transformed reproductive hormones using linear regressions is presented in Table 3. Initially, in the unadjusted univariate analyses a positive association between serum vitamin B_12_ concentrations and TT (β = 0.0007, *P* = 0.008) was observed. In the adjusted multivariate model, the positive relationship between serum vitamin B_12_ concentrations and TT remained significant (β = 0.0005, *P* = 0.03). No other statistically significant associations were observed between serum vitamin B_12_ concentrations and reproductive hormones (LH, FSH, prolactin, and estradiol) in both univariate and multivariate linear regression analyses. In covariate-adjusted linear regression analyses, the potential effect modification of age and BMI was explored. In the regression analyses with inclusion of age as an effect modifier, there was no significant interaction between age and vitamin B_12_ concentrations. In similar analyses, but with the inclusion of BMI as an effect modifier, there was also no significant interaction between BMI and serum vitamin B_12_ concentrations. Since there were no significant interactions for effect modification, simple slope and false discovery rate-corrected Johnson–Neyman interval analyses were not conducted. Thus, both age and BMI did not modify the linear relationship between serum vitamin B_12_ concentrations and reproductive hormones. Results for effect modification analyses are presented in Table 3.

**TABLE 3:** Beta-coefficient (± SE) and corresponding *P* for the linear association between serum vitamin B_12_ concentration (pmol/L) and serum reproductive hormones concentrations.

Reproductive hormones^1^	Unadjusted model β ± SE	Unadjusted *P*	Adjusted model β ± SE^2^	Adjusted *P*	Interaction by age *P*^3^	Interaction BMI *P*^4^
FSH	−0.0005 ± 0.0004	0.20	−0.0004 ± 0.0004	0.33	0.51	0.56
LH	−0.0004 ± 0.0002	0.11	−0.0003 ± 0.0002	0.21	0.15	0.42
TT	0.0007 ± 0.0002	0.008	0.0005 ± 0.0002	0.03	0.68	0.48
Prolactin	0.00002 ± 0.00005	0.65	0.00003 ± 0.00005	0.59	0.84	0.79
Estradiol^5^	0.0003 ± 0.0007	0.61	0.0003 ± 0.0007	0.71	0.16	0.35

Abbreviations: BMI, body mass index; FSH, follicular stimulating hormone; LH, luteinizing hormone; SE, standard error; TT, total testosterone.

1 Reproductive hormones (FSH, LH, TT, and prolactin) are square root transformed. The relationship depicted is the association between serum ascorbic acid concentration and square root of reproductive hormones (FSH, LH, TT, and prolactin).

2 Model adjusted for covariates: age, alcohol consumption, BMI, ethnicity, seasonal variation, and smoking status.

3 Interaction *P* for the adjusted linear regression model assessing the association between serum vitamin B_12_ concentration and serum reproductive hormones with effect modification by age (age by vitamin B_12_ interaction term).

4 Interaction *P* for the adjusted linear regression model assessing the association between serum vitamin B_12_ concentration and serum reproductive hormones with effect modification by BMI (BMI by vitamin B_12_ interaction term).

5 Estradiol was categorized into groups: normal (≤146.8 pmol/L) and elevated (>146.8 pmol/L).

Results from the logistic regression analyses assessing the association between tertiles of vitamin B_12_ concentrations (with the lowest tertile as the reference group) and the clinical status of reproductive hormones (with normal hormone concentrations as the reference group) are presented in Table 4 and Supplemental Figure 2. For LH, in the unadjusted analyses, individuals in the highest tertile of serum vitamin B_12_ concentrations had reduced odds of elevated LH compared with those in the lowest tertile of serum vitamin B_12_ concentrations (odds ratio [OR] = 0.50; 95% confidence interval [CI]: 0.28, 0.90, *P* = 0.02). However, the association was no longer significant in the adjusted model (OR = 0.53; 95% CI: 0.25, 1.13, *P* = 0.05). Compared with individuals in the lowest tertile of serum vitamin B_12_ concentration, there was a lower odds of TT deficiency among those in the mid-tertile (OR = 0.48; 95% CI: 0.25, 0.93, *P* = 0.03) and upper tertile (OR = 0.44; 95% CI: 0.22, 0.87, *P* = 0.02) of serum vitamin B_12_ concentration. For all other remaining reproductive hormones, in both unadjusted and adjusted analyses, there were no significant differences in the odds of experiencing hormone concentrations outside the normal range based on the tertiles of serum vitamin B_12_ concentrations. Prolactin was not included in the logistic regression analyses due to minimal variability (<10%) in elevated prolactin concentrations among the study participants.

**TABLE 4:** Odds ratios (ORs) and 95% confidence intervals (CIs) for the association between tertiles of serum vitamin B_12_ concentration and reproductive hormones outside of normal range^1^ using binomial logistic regressions.

Hormone	Serum vitamin B_12_ mid-tertile^2^				Serum vitamin B_12_ highest tertile^3^			
	Unadjusted OR [95% CI]	Unadjusted *P*	Adjusted OR [95% CI]^4^	Adjusted *P*	Unadjusted OR [95% CI]	Unadjusted *P*	Adjusted OR [95% CI]^4^	Adjusted *P*
FSH	0.61 [0.32, 1.15]	0.13	0.53 [0.27, 1.06]	0.07	0.68 [0.36, 1.27]	0.22	0.75 [0.37, 1.50]	0.40
LH	0.65 [0.36, 1.15]	0.14	0.61 [0.29, 1.25]	0.10	0.50 [0.28, 0.90]	0.02	0.53 [0.25, 1.13]	0.05
TT	0.58 [0.32, 1.04]	0.07	0.48 [0.25, 0.93]	0.03	0.41 [0.22, 0.77]	0.005	0.44 [0.22, 0.87]	0.02
Estradiol	1.26 [0.72, 2.36]	0.48	1.43 [0.73, 2.84]	0.30	1.35 [0.92, 2.54]	0.36	1.2 [0.60, 2.40]	0.61

Abbreviations: BMI, body mass index; CI, confidence interval; FSH, follicular stimulating hormone; LH, luteinizing hormone; OR, odds ratio; TT, total testosterone.

1 The clinical status cutoffs were determined based on reproductive hormones outside of normal range: elevated FSH (>12.4 IU/L), elevated LH (>7.8 IU/L), low TT (<9.2 nmol/L), and elevated estradiol (>146.8 pmol/L). Serum reproductive hormones within the normal range and first tertile of serum vitamin B_12_ concentration were used as the reference category for all analyses.

2 ORs compare the odds of experiencing reproductive hormones outside of normal range for participants in the mid-tertile of serum vitamin B_12_ concentration (>340 to <472.7 pmol/L) with participants in the lowest tertile (<340 pmol/L) of serum vitamin B_12_ concentration.

3 ORs compare the odds of experiencing reproductive hormones outside of normal range for participants in the highest tertile (≥472.7 pmol/L) of serum vitamin B_12_ concentration with the odds for those in the lowest tertile of serum vitamin B_12_ (≤340 pmol/L) concentration.

4 Model adjusted for covariates: age, alcohol consumption, BMI, ethnicity, seasonal variation, and smoking status.

## Discussion

A few studies have examined the association between serum vitamin B_12_ concentrations and male reproductive hormones. Our findings show a positive linear association between vitamin B_12_ and serum TT concentrations. In a recent study investigating the effects of ascorbic acid on reproductive hormones, an age-dependent effect of ascorbic acid on TT was found [36]. However, in the present study, we did not observe any modifications in the linear relationship between serum vitamin B_12_ and TT based on age or BMI. Additionally, we discovered that individuals in either the mid- or highest tertiles of serum vitamin B_12_ had significantly lower odds of experiencing testosterone deficiency compared with those in the lowest tertile of vitamin B_12_. Although the highest tertile showed the lowest odds, it is noteworthy that even individuals in the mid-tertile experienced a significant reduction in the odds of TT deficiency. This suggests that even a slight increase in vitamin B_12_ concentrations may contribute to a notable decrease in the likelihood of testosterone deficiency. Although vitamin B_12_ showed a significant positive linear relationship with TT concentrations, there was no evidence of a similar association with the other reproductive hormones (FSH, LH, prolactin, and estradiol). Furthermore, there was no significant association between tertiles of serum vitamin B_12_ and the odds of experiencing elevated concentrations of FSH, LH, and estradiol. These results suggest that the influence of vitamin B_12_ on hormonal regulation may be specific to testosterone.

To our knowledge, only one previous study examined the association between vitamin B_12_ administration and male reproductive hormones [35]. Isoyama et al. [35] conducted a clinical trial with a group of 26 infertile males and found daily administration of 1500 μg of methylcobalamin for 4–24 wk resulted in some improvements in sperm parameters but no changes in serum FSH, LH, or testosterone. However, that study had several limitations, including the absence of a control group, a limited sample size (*n* = 26), a variable intervention duration (4–24 wk), no measurement or reporting of serum vitamin B_12_ concentration, lack of measurement of compliance to B_12_ supplementation, and no consideration for dietary vitamin B_12_ intake [35]. In addition, there was no assessment of baseline vitamin B_12_ concentrations. Without information on baseline vitamin B_12_ status, supplementation may not have any discernible impact, especially if individuals already had elevated B_12_ concentrations at baseline [35].

A previous study compared the effects of vitamin B_12_ and methotrexate administration, alone or in combination, on hormone concentrations in rodents [37]. In comparison with the control group, testosterone concentrations increased, but FSH and LH concentrations remained unchanged after administration of vitamin B_12_ [37]. This is similar to the present study’s findings where a linear relationship between serum vitamin B_12_ and testosterone was found, but no significant relationship between serum vitamin B_12_ and either LH or FSH was found. Their findings suggest that vitamin B_12_ may have a protective effect by reducing apoptosis and alleviating endoplasmic reticulum stress associated with testicular toxicity as vitamin B_12_ supplementation resulted in improved hormone concentrations, and supported the production of normal germ cells even in the presence of normal hormone concentrations [37].

Despite previous research suggesting men with infertility may have lower serum vitamin B_12_ concentrations compared to men who do not experience infertility, the prevalence of vitamin B_12_ deficiency was low in the current study population at 0.7% [38,39]. This is lower than the 1.2% prevalence of vitamin B_12_ deficiency reported in the Canadian general population by the Canadian Health Measures Survey [40]. However, a substantial number of the study participants, specifically 12.2%, exhibited elevated serum vitamin B_12_ concentrations.

The present study has several strengths. It is the first comprehensive investigation exploring the relationship between serum vitamin B_12_ and several key male reproductive hormones in humans. Furthermore, clinically significant covariates were predefined, rather than employing a data-driven approach. Additionally, these covariates were accounted for in statistical analyses, and the utilization of a nutrient biomarker minimized potential self-reporting biases associated with dietary intake assessments. However, certain limitations should be acknowledged. Given the cross-sectional nature of the study design, establishing causality and temporality is not possible. Furthermore, all demographic and anthropometric data relied on self-reporting, introducing a potential risk of self-reporting bias for some of the covariates. In addition, the present study was designed to examine the independent association between vitamin B_12_ and reproductive hormones. However, the combined influence of several micronutrients and macronutrients could potentially collectively influence reproductive hormones differently. Although several critical factors were adjusted for in the analyses, unidentified factors that were not adjusted for in the analyses could also potentially influence our findings.

The present study contributes new insights by enhancing the current understanding of the potential impact of vitamin B_12_ on male reproductive hormones in men with infertility. It provides a foundation for future investigations in the broader general population, in older age male cohorts, and for clinical trials exploring vitamin B_12_ supplementation for men. The findings have potential clinical significance where the standard B_12_ reference values may need to be re-examined to consider fertility. Our findings also suggest that B_12_ concentrations may need to be assessed as part of routine measures in the management of male infertility. Enhanced knowledge in this field holds potential significant benefits, such as incorporating nutritional assessments and interventions with minimal side effects into male infertility management, including supporting more successful outcomes from assisted reproductive technologies.

## Acknowledgments

We would like to thank Irtaza Tahir, Konrad Samsel, Matthew Pasquini, and Susan Lau for their support and assistance during the research study.

## Author contributions

The authors’ responsibilities were as follows – MRP, KJ, and AE-S designed the research; MP conducted research, analyzed data, and wrote the article. AE-S had primary responsibility for final content; and all authors: read and approved the final manuscript.

## Conflicts of interest

AE-S holds shares in Nutrigenomix Inc., which provides genetic testing for personalized nutrition. The remaining authors declare that the research was conducted in the absence of any commercial or financial relationships that could influence the study.

## Funding

The research was funded by the Canadian Federation for Dietetic Research and the Allen Foundation Inc.

## Data availability

Data described in the manuscript, code book, and analytic code will be made available upon request pending application and approval.
