# Supplementary material for: Vitamin B12 Is Associated with Higher Serum Testosterone Concentrations and Improved Androgenic Profiles Among Men with Infertility
Source: J Nutr. 2024 Jun 26;154(9):2680–7. doi: 10.1016/j.tjnut.2024.06.013 (PMC11393164; doi:10.1016/j.tjnut.2024.06.013)
Supplement: Multimedia component 1 [file mmc1.docx]

**Supplementary Figure 1: Participant flow chart**

**Total participants recruited for nutrition and fertility study:** 832

**Excluded Participants:**

Missing nutritional bloodwork and hormonal assay (*n* = 476)

Missing lifestyle, general health, and anthropometric data (*n* = 44)

Klinefelter Syndrome, cystic fibrosis, vasectomy, Y chromosome microdeletions and < 4 years previous testicular cancer radiation therapy (*n* = 9)

**Total Sample Size:** 303

**Supplementary Figure 2:** Forest plot representing the odds and 95% Confidence Intervals (CIs) for experiencing reproductive hormones outside of normal range ^1^ in the highest and mid- verses lowest tertile of serum vitamin B_12_ concentration after covariate adjustments ^2^

Mid-tertile ^3^

Highest tertile ^4^

FSH

LH

TT

Estradiol

Odds Ratio

| ^1^ Reproductive hormones outside of normal range defined as, elevated FSH (> 12.4 IU/L), elevated LH (> 7.8 IU/L), low TT (< 9.2 nmol/L) and elevated estradiol (> 146.8 pmol/L). Serum reproductive hormones within the normal range were used as the reference category for all analyses.  ^2^ Model adjusted for: age, alcohol consumption, BMI, ethnicity, seasonal variation, and smoking status.  ^3^ ORs compare the odds of experiencing reproductive hormones outside of normal range for participants in the mid-tertile of serum vitamin B_12_ concentration (> 340 pmol/L to < 472.7 pmol/L) with participants in the lowest tertile (< 340 pmol/L) of serum vitamin B_12_ concentration.  ^4^ ORs compare the odds of experiencing reproductive hormones outside of normal range for participants in the highest tertile (> 472.7 pmol/L) of serum vitamin B_12_ concentration with the odds for those in the lowest tertile of serum vitamin B_12_ (< 340 pmol/L) concentration. |
| --- |
